# Spectral data of tropical soils using dry-chemistry techniques (VNIR, XRF, and LIBS): A dataset for soil fertility prediction

**DOI:** 10.1016/j.dib.2022.108004

**Published:** 2022-03-01

**Authors:** Tiago Rodrigues Tavares, José Paulo Molin, Lidiane Cristina Nunes, Elton Eduardo Novais Alves, Francisco José Krug, Hudson Wallace Pereira de Carvalho

**Affiliations:** a Department of Biosystems Engineering, “Luiz de Queiroz” College of Agriculture (ESALQ), University of São Paulo (USP), Piracicaba, São Paulo 13418900, Brazil; b Center for Nuclear Energy in Agriculture (CENA), University of São Paulo (USP), Piracicaba, São Paulo 13416000, Brazil; c Department of Chemistry, Federal University of Minas Gerais, Belo Horizonte, Minas Gerais, 31270-901, Brazil; d CESFRA, AgroBioSciences, Mohammed VI Polytechnic University, BenGuerir 43150, Morocco

**Keywords:** Soil spectral libraries, Proximal Soil Sensing, Precision agriculture, Hybrid laboratory, Pedometrics

## Abstract

Proximal soil sensing technologies, such as visible and near infrared diffuse reflectance spectroscopy (VNIR), X-ray fluorescence spectroscopy (XRF), and laser-induced breakdown spectroscopy (LIBS), are dry-chemistry techniques that enable rapid and environmentally friendly soil fertility analyses. The application of XRF and LIBS sensors in an individual or combined manner for soil fertility prediction is quite recent, especially in tropical soils. The shared dataset presents spectral data of VNIR, XRF, and LIBS sensors, even as the characterization of key soil fertility attributes (clay, organic matter, cation exchange capacity, pH, base saturation, and exchangeable P, K, Ca, and Mg) of 102 soil samples. The samples were obtained from two Brazilian agricultural areas and have a wide variation of chemical and textural attributes. This is a pioneer dataset of tropical soils, with potential to be reused for comparative studies with other datasets, *e.g.*, comparing the performance of sensors, instrumental conditions, and/or predictive models on different soil types, soil origin, concentration range, and agricultural practices. Moreover, it can also be applied to compose soil spectral libraries that use spectral data collected under similar instrumental conditions.

## Specifications Table

**Table:** 

Subject	Soil Science.
Specific subject area	Proximal soil sensing, soil fertility analysis.
Type of data	Tables (.txt, tab delimited).
How data were acquired	VNIR data (from 431.59 to 2153.11 nm, with 351 data points) were acquired using the spectrometer Veris MSP3 (Veris Technologies, Salina, Kansas, USA); XRF data (from 0.01 to 40.74 keV, with 2048 data points) were acquired with the portable device Tracer III-SD model (Bruker AXS, Madison, Wisconsin, EUA); LIBS data (from 200.005 to 779.992 nm, with 53717 data points) were acquired using a benchtop system composed by a pulsed Nd:YAG laser at 1064 nm, generating 5 ns pulses of up to 365 mJ (Brilliant, Quantel, France) and an ESA 3000 spectrometer (LLA Instruments GmbH, Berlin, Germany); Soil fertility analyses for determining clay, organic matter (OM), cation exchange capacity (CEC), pH, base saturation (V), exchangeable (ex-) P, ex-K, ex-Ca, and ex-Mg were performed in a commercial laboratory.
Data format	Raw.
Parameters for data collection	The VNIR data was acquired after the spectrometer calibrates itself using reference materials with known spectral behaviour; For XRF data acquisition the X-ray tube was set for voltage and current of 35 kV and 7 µA, respectively. No vacuum condition or filters were used for the XRF spectra acquisition; For LIBS data acquisition, the following instrumental conditions were used: 225 J cm^−2^ (65 mJ per pulse at 180 µm laser spot size), 15 accumulated laser pulses per site, 2.0 µs of delay time, and 7.0 µs of integration time gate.
Description of data collection	Soil samples were collected from 0 to 20 cm depth; Loose powder soil samples (dry and grain size < 2 mm) were analysed with the VNIR and XRF sensor. For LIBS data acquisition, the samples were pelletized after being comminuted in a planetary ball mill with a binder material; Spectral data acquisition was performed under laboratory conditions; For VNIR and XRF data collection, each sample was scanned in triplicate repositioning the sample after each scan. For LIBS data collection, the pressed pellets were sampled at 21 different sites, thus yielding 21 optical emission spectra. Approximately 1 mm distance was kept between sites to avoid re-ablation at the edges of the neighboring craters. For all techniques, the analytical signals of the replicates were averaged in one reading, which represents the spectral response of each sample.
Data source location	Soil samples were collected from two agricultural fields, as described below. City/Region: municipality of Piracicaba, State of São Paulo (Field 1) and municipality of Campo Novo do Parecis, State of Mato Grosso (Field 2). Country: Brazil. Latitude and longitude: 22°41′57″ S and 47°38′33″ W, for Field 1, and 14°06′ 05″ S and 57°45′58″ W, for Filed 2 (both referred using WGS-84 datum).
Data accessibility	Repository name: “Spectral data of tropical soils using dry-chemistry techniques (VNIR, XRF, and LIBS): a dataset for soil fertility prediction” Data identification number: doi:10.17632/88c5kvmgbf.1 Direct URL to data: https://data.mendeley.com/datasets/88c5kvmgbf/1 This study was exempt from an ethic approval process because it does not involve hazards and human or animal subjects.
Related research article	T.R. Tavares, J.P. Molin, L.C. Nunes, M.C.F. Wei, F.J. Krug, H.W.P. Carvalho, A.M. Mouazen, Multi-Sensor Approach for Tropical Soil Fertility Analysis: Comparison of Individual and Combined Performance of VNIR, XRF, and LIBS Spectroscopies, Agronomy 11 (2021) 1028. https://doi.org/10.3390/agronomy11061028

## Value of the Data

• The techniques applied for data acquisition allow rapid, non-destructive, and reagent-free analysis; studies involving these techniques are incipient in the context of proximal soil sensing and this database is among the pioneers in tropical soils.
• This database can be used for comparative studies with other datasets, e.g., comparing the performance of sensors, instrumental conditions, and/or predictive models on with different soil types, soil origin, concentration range, and agricultural practices.
• This database can also be used to evaluate predictive models little explored in the literature to exploit the synergies among sensors.
• This database also could be used to compose soil spectral libraries that use data collected under similar instrumental conditions.

## 1 Data Description

**Fig. 1:**
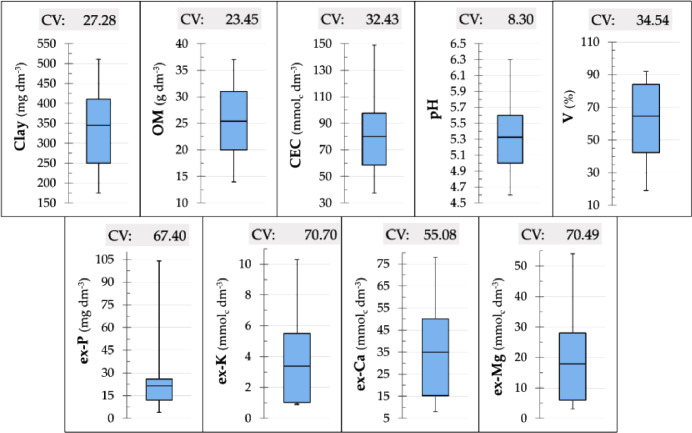
Boxplot of the clay, organic matter (OM), cation exchange capacity (CEC), pH, base saturation (V), and extractable (ex-) P, K, Ca, and Mg content (*n* = 102 soil sample from Field 1 and 2), which are the soil fertility attributes to be used as Y-variables in predictive modelling. The coefficient of variation (CV) for each attribute was also shown and expressed in %. This figure was modified from Tavares et al [5].

**Fig. 2:**
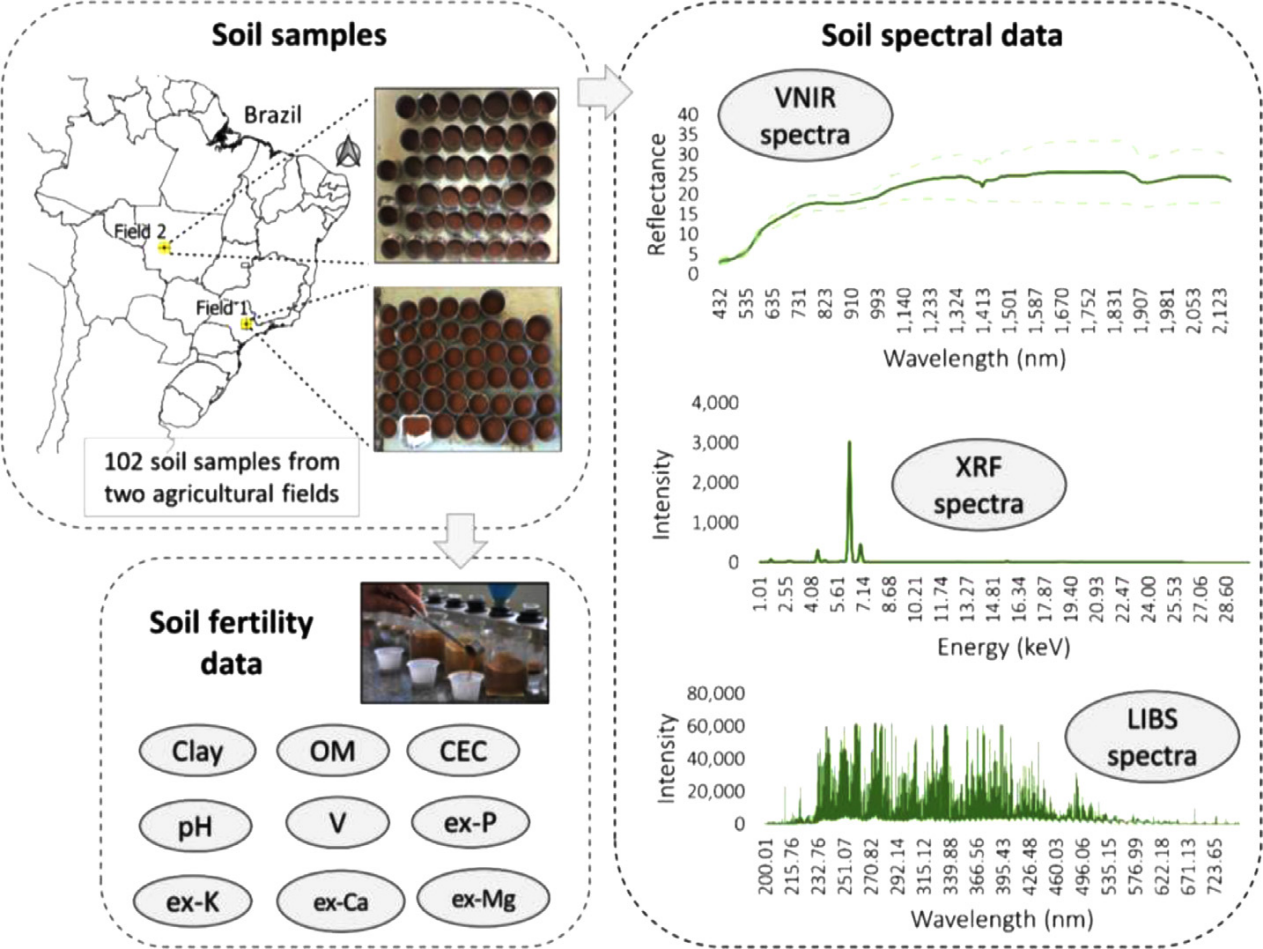
Framework of the shared dataset. In this study, 102 soil samples were collected from tropical agricultural fields were scanned using visible and near infrared diffuse reflectance spectroscopy (VNIR), X-ray fluorescence spectroscopy (XRF), and laser-induced breakdown spectroscopy (LIBS), and also sent to a commercial laboratory for determining clay, organic matter (OM), cation exchange capacity (CEC), pH, base saturation (V), and extractable (ex-) P, K, Ca, and Mg content. Soil spectra can be used as X-variables and soil fertility attributes as Y-variables in predictive modelling. This figure was modified from Tavares et al [5].

The dataset contains spectral data and characterizations of key soil fertility attributes of 102 soil samples. These samples are from two Brazilian agricultural areas, which have soils classified as Lixisol (Field 1) and Ferralsol (Field 2) [1]. Both type of soils are commonly found in Brazil's tropical regions [2], as well as in tropical regions of Africa, Asia, and Oceania [1]. Agricultural areas in the Brazilian tropical region are generally acidic and have low natural fertility, this characteristic makes soil fertility analysis fundamental for the correct prescription of fertilizers [3]. It is estimated that about 7 million samples are analysed annually in traditional analytical laboratories; furthermore, Brazil is the fourth largest consumer of fertilizers in the world [4]. The chosen fields have different soil matrices due to considerable textural and total elemental composition contrast. Regarding the fertility attributes, the two-field dataset present wide ranges of the variability of fertility attributes, as shown in Fig. 1. After soil fertility tests, the samples were scanned with the following direct analysis techniques: (i) visible and near infrared diffuse reflectance spectroscopy (VNIR), (ii) X-ray fluorescence spectroscopy (XRF), and (iii) laser-induced breakdown spectroscopy (LIBS). The components of the shared dataset are schematized in Fig. 2.

The shared dataset contains four tables (which were shared in both .txt and .xlsx format) named as ``soil fertility data'', ``VNIR data'', ``XRF data'', and ``LIBS data'', which respectively contain the data from the soil fertility analysis, VNIR, XRF, and LIBS spectroscopies. The tables/datasets ``soil fertility data'', ``VNIR data'', and ``XRF data'' are organized in dataframes with long format (*i.e.*, observations in rows and variables in columns) and the table containing the LIBS data is a dataframe with wide format (*i.e.,* observations in columns and variables in rows). All datasets have 102 observations and have as primary key the variable ID (first variable of all datasets), which identifies the samples (observations) with sequential numbers. The second variable of all datasets is named ``Field'' and contains the category “1”, for samples from Field 1 (*n* = 58), and the category “2”, for samples from Field 2 (*n* = 44). The other variables of each dataset are specified below.

• ``Soil fertility data'': from column 3 to 11 (9 variables) are the contents of clay, organic matter (OM), cation exchange capacity (CEC), pH, base saturation (V), exchangeable (ex-) P, ex-K, ex-Ca, and ex-Mg, respectively. The values are given in g dm^−3^ for clay and OM; in mmol_c_ dm^−3^ for CEC, ex-K, ex-Ca, and ex-Mg; in % for V; and, for ex-P, it was given in mg dm^−3^.
• ``VNIR data'': from column 3 to 353 (351 variables) are the reflectance values (expressed in %) of wavelengths ranging from 431.59 to 2153.11 nm.
• ``XRF data'': from column 3 to 2050 (2048 variables) are the emission intensity values (in counts of photons per second) of energies oscillating from 0.01 to 40.74 keV.
• ``LIBS data'': from row 3 to 53719 (53717 variables) are the emission intensity values (in arbitrary unit) of wavelengths ranging from 200.005 to 779.992 nm.

## 2 Experimental Design, Materials and Methods

### 2.1 Soil samples, fertility analysis, and sample preparation for spectroscopic analyses

A total of 58 samples were collected in the Field 1, which is located in the municipality of Piracicaba, State of São Paulo, Brazil. The remaining samples (*n* = 44) were collected in the Field 2, situated in the municipality of Campo Novo do Parecis, State of Mato Grosso, Brazil. All samples were collected from 0 to 20 cm depth. The soil samples were subjected to laboratory analyses, which provided the contents of clay, OM, CEC, pH, V, ex-P, ex-K, ex-Ca, and ex-Mg. These determinations followed the methods described by Van Raij et al [6], in which, clay was determined by using the Bouyoucos hydrometer method; extractable nutrients by using ion exchange resin extraction; OM by oxidation with potassium dichromate solution, and pH by calcium chloride solution. The CEC was calculated by the sum of the soil potential acidity and the sum of bases (ex-K + ex-Ca + ex-Mg); in turn, the soil potential acidity determined via buffer solution (SMP). The V was also calculated and represents the percentage of bases in the CEC.

For VNIR and XRF data acquisition, it was used loose powder soil samples (air-dry and grain size ≤ 2 mm). Whereas, for LIBS data acquisition, it was used pelletized samples. For pelletizing, samples were comminuted in a planetary ball mill with a 10% w w^−1^ binder material (microcrystalline cellulose, Merck, Darmstadt, Germany)and then pressed with a press, as detailed by Tavares et al [7].

### 2.2 VNIR data acquisition

The device Veris MSP3 (Veris Technologies, Salina, Kansas, USA) was used for VNIR data acquisition. This system consists of a tungsten halogen lamp, as energy source, and a detection system composed by two spectrometers: (i) a CCD array (USB4000, Ocean optics, Largo, FL, USA) and (ii) an InGaAs photodiode-array (C9914GB, Hamamatsu Photonics, Hamamatsu, Japan). This spectrometer set allows to record the spectra from 343.00 to 2222.00 nm, with spectral resolution of ±5 nm. The VNIR spectrometer automatically checks the measured reflectance behaviour using four references materials with known spectral behaviour. In addition, it was self-calibrated, by making a dark and white reference measurements, before each spectra acquisition. The sample holder isolates the sample from ambient light. Each sample was scanned in triplicate, by repositioning the sample after each reading, and then the replicates were averaged. The spectra edges (from 343.00 to 431.59 nm and from 2153.11 to 2222.00 nm) were removed due to the high presence of noise.

### 2.3 XRF data acquisition

A portable energy dispersive X-ray fluorescence spectrometer, Tracer III-SD model (Bruker AXS, Madison, Wisconsin, EUA), equipped with a 4 W Rh X-ray tube and a Peltier-cooled Silicon Drift Detector (with 2048 channels, gain of ∼20 eV/channel) was used for XRF data acquisition. The following instrumental conditions were used: (i) X-ray tube voltage of 35 kV and current of 7 µA; (ii) dwell time of 90 s; (iii) no filter was used; and (iv) scans were performed under atmospheric pressure. Three measurements were taken from each soil sample at three different spots, and these were then averaged.

### 2.4 LIBS data acquisition

For LIBS data acquisition, it was used a benchtop LIBS system composed by a pulsed Nd:YAG laser (Brilliant, Quantel, France) and an ESA 3000 spectrometer (LLA Instruments GmbH, Berlin, Germany). The laser operates at 1064 nm, generating 5 ns pulses of up to 365 mJ, in a 6 mm diameter beam, at 10 Hz repetition rate. The laser pulse was focused on the sample surface by a plane-convex lens with 2.54 cm diameter and 20 cm focal length. Pressed pellets were placed into a plastic sample holder positioned in a two-axes manually-controlled translation stage, movable in the plane orthogonal to the laser direction. A laminar stream of argon (5.0 L min^−1^) was continuously fed from the bottom of the sample holder in order to dislocate the atmospheric air around the sample surface. The emission from the plasma was collected by using a telescope (positioned about 25° from the laser axis) composed of 50 and 80 mm focal length fused silica lenses and coupled to the entrance slit of the spectrometer using an optical fiber. The spectrometer device is equipped with Echelle optics (focal length of 25 cm with numerical aperture of 1:10) and an ICCD camera detector that is comprised of a Kodak KAF 1001 CCD array of 1024 × 1024 pixels full frame, enabling registers spectra from 200 to 780 nm with a resolution oscillating from 5 pm at 200 nm to 19 pm at 780 nm.

LIBS instrumental conditions were optimized in initial tests to obtain the maximum signal-to-noise ratio of the emission lines of interest, as suggested by Nunes et al [8]. The experimental conditions used for data acquisition were: 65 mJ laser pulses, 225 J cm^−2^ (65 mJ per pulse at 180 µm laser spot size, 19.5 cm of lens-to-sample distance), 15 accumulated laser pulses per site, 2.0 µs of delay time, and 7.0 µs of integration time gate. The pressed pellets were sampled at 21 different sites in order to consider the analytes micro-heterogeneity in the samples; then, the replicates were averaged.

## Ethics Statement

The authors declare that the work does not involve the use of human subjects, animal experiments, or data collected from social media platforms, being exempt from an ethic approval process.

## CRediT authorship contribution statement

**Tiago Rodrigues Tavares:** Conceptualization, Funding acquisition, Investigation, Methodology, Data curation, Formal analysis, Writing – original draft. **José Paulo Molin:** Conceptualization, Funding acquisition, Supervision, Validation, Writing – review & editing. **Lidiane Cristina Nunes:** Methodology, Data curation, Formal analysis, Writing – review & editing. **Elton Eduardo Novais Alves:** Methodology, Data curation, Formal analysis, Writing – review & editing. **Francisco José Krug:** Supervision, Validation, Writing – review & editing. **Hudson Wallace Pereira de Carvalho:** Supervision, Validation, Writing – review & editing.

## Declaration of Competing Interest

The authors declare that they have no known competing financial interests or personal relationships which have or could be perceived to have influenced the work reported in this article.

## Data Availability

Spectral data of tropical soils using dry-chemistry techniques (VNIR, XRF, and LIBS): a dataset for soil fertility prediction (Original data) (Mendeley Data Repository).
